# Effect of Veneering and Hydrothermal Aging on the Translucency of Newly Introduced Extra Translucent and High Translucent Zirconia with Different Thicknesses

**DOI:** 10.1155/2021/7011021

**Published:** 2021-10-21

**Authors:** Sevki Cinar, Bike Altan

**Affiliations:** ^1^Department of Prosthodontics, Faculty of Dentistry, University of Health Sciences, Tıbbiye Street No. 38, 34668, Uskudar, Istanbul, Turkey; ^2^Vocational School of Health Services, University of Health Sciences, Uskudar, Istanbul, Turkey

## Abstract

**Purpose:**

The purpose of this study was to evaluate the effect of veneering and aging on the translucency of newly introduced extra and high translucent zirconia with different thickness.

**Materials and Methods:**

One hundred forty disk-shaped specimens were fabricated from two translucent zirconia blocks (VITA YZ XT and VITA YZ HT), and they are milled with CAD/CAM system. Then, specimens were divided into nonveneered (XT, HT) and veneered groups (XTV, HTV). Nonveneered groups were prepared with four different thicknesses (0.5-1-1.5-2 mm). Veneered groups were divided into three subgroups (*n* = 10) for veneering with base dentin ceramic with thicknesses of 0.5 + 0.5, 0.5 + 1, and 0.5 + 1.5 mm. A spectrophotometer was used to calculate the translucency parameter (TP) and contrast ratio (CR) of all specimens before and after aging. Statistical analysis was performed using 3-way ANOVA and Tukey HSD tests (*p* < 0.05).

**Results:**

TP values were significantly affected by thickness of specimens (*p* < 0.001). VITA YZ XT was significantly found more translucent than VITA YZ HT. The highest translucency was observed in the XT-0.5 mm group. There is no significant difference between translucency of the veneered and nonveneered groups in the same thickness for XT. On the contrary, veneering significantly affected translucency of HT. TP values significantly decreased after aging for all groups. After aging, translucency value difference before and after aging was the highest in the XT-0.5 mm group whereas the HTV-2 mm group showed the lowest difference after aging. TP decreased significantly as thickness of specimen increases regardless of the material type. Extra translucent and nonveneered zirconia groups are more prone to hydrothermal aging.

**Conclusions:**

The translucency parameter of zirconia ceramics was significantly influenced by both material type and veneering. Also, extra translucent and nonveneered zirconia groups are more susceptible to hydrothermal aging.

## 1. Introduction

Increased demand of patients has led to the increase in use of zirconia restorations compared to porcelain-fused-to-metal (PFM) restorations. Zirconia restorations gained attention thanks to their biocompatibility, high fracture strength, and esthetic properties [1]. Conventional Y-TZP zirconia restorations are often used with veneering ceramic due to their opaque nature, which leads to chipping and delamination [2]. To overcome this issue, monolithic zirconia is developed. Monolithic zirconia is a high translucent material with decreased alumina content compared to conventional zirconia and characterized by cubic phase and low porosity [3]. In addition, recently extra translucent monolithic zirconia block is commercially available. However, much more translucency may be required to mimic a natural tooth, and therefore, monolithic zirconia may be veneered with feldspathic ceramics [4].

Monolithic zirconia restorations have been increasingly used due to their esthetics and high translucency. Recently, extra translucent zirconia which has isotropic crystal structure has been introduced into dentistry [5]. Clinicians demand more translucence, and natural restorations and veneering may provide to give restorations a more lifelike appearance and characterization. Veneering with ceramics also helps match the texture of natural adjacent teeth [2]. Therefore, the goal of the study was to investigate the translucency of these materials. Few data are available on translucency of newly introduced extra and high translucent zirconia blocks, and no studies directly compared optical properties of veneered and nonveneered restorations of these materials.

Translucency has been considered as one of the primary factors in maintaining esthetics and so that is important for material selection [6]. Translucency can be defined as the relative amount of light transmission or diffuse reflectance from a substrate surface through a turbid medium [7]. Light transmittance plays an important role in designing lifelike and natural restorations. If light is intensely scattered and diffusely reflected, the material will appear opaque, and if only part of the light is scattered and most is diffusely transmitted, it will appear translucent [8].

Translucency of zirconia is dependent on thickness [9, 10], grain size [11], phase content [2], sintering temperature [11–13], porosity [14], and aging [15, 16]. Optically isotropic cubic phase reduced birefringence effect and makes zirconia more translucent [2, 17]. Besides, larger grain size makes material more compact by means of higher sintering temperature and porosity elimination and thus increases translucency [18]. The larger the particles, the smaller the number of grain boundaries where the light is refracted [19].

On the other hand, large grains are more prone to hydrothermal degradation [20]. Tetragonal to monoclinic phase transformation (LTD) occurs in oral environment during the lifetime of zirconia restorations. Low-temperature degradation of zirconia is simulated by steam autoclave at increased temperatures. Recent studies showed that the translucency of zirconia ceramics is affected by hydrothermal aging [16, 17, 21].

The translucency of a material is usually determined using the translucency parameter (TP) and contrast ratio (CR). TP is defined as the color difference between a uniform thickness of a material over a white and a black backing. CR is defined as the ratio of illuminance (*Y*) of the test material when placed over a black background to the illuminance of the same material when placed over a white background [22].

The aim of this study is to investigate the optical properties of veneered and nonveneered translucent zirconia which have different translucency values with different thicknesses. Additionally, the study is aimed at comparing the translucency of two translucent zirconia blocks before and after artificial aging. The null hypothesis was that translucency of zirconia is not affected by the type of material, thickness, and artificial aging.

## 2. Materials and Methods

**Figure 1:**
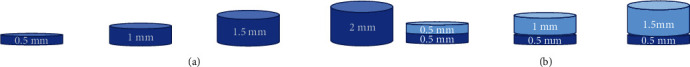
Size and form of specimens. (a) Nonveneered (XT, HT) and (b) veneered (XTV, HTV) groups. As light-colored ones present zirconia, dark-colored ones present veneering ceramics.

A total of 140 disk-shaped specimens (14 mm in diameter) of color A2 were fabricated from two translucent zirconia blocks (VITA YZ XT and VITA YZ HT, Vita Zahnfabrik, Germany) milled with CAD/CAM system (Cerec inLab, Dentsply Sirona, York, PA). Chemical composition of the materials is shown in Table 1. Specimens were divided into the nonveneered (XT, HT) and veneered (XTV, HTV) groups. The nonveneered groups were prepared with four different thicknesses (0.5, 1, 1.5, and 2 mm) as shown in Figure 1. Specimen thickness was adjusted to compensate the shrinkage of zirconia during sintering process and then measured with a digital micrometer (Mitutoyo, Japan, Kanagawa). Then, specimens were sintered at 1450°C for 4:40 hours according to manufacturer instructions (VITA Zyrcomat 6000 MS). The veneered groups (HTV and XTV) were divided into three subgroups (*n* = 10) for veneering with base dentin ceramic (VITA VM 9, Vita Zahnfabrik, Germany) with thicknesses of 0.5, 1, and 1.5 mm. The shades for the veneer ceramics were selected to correspond to A2 shade. Dentin ceramics were prepared with custom-made molds which were used for standardizing the dentin thickness.

Then, firing process was performed at 910°C in the furnace (VITA Vacumat 6000 M). Then, veneered specimens were ground and polished on a device (Phoenix Beta; Buehler, USA) using 400 to 2400 grit SiC papers whereas nonveneered specimens were polished with 1200 to 2400 grit SiC papers (English Abrasives). The final thickness of specimens (zirconia plus veneering ceramic) was set to 0.5 (no veneering), 1, 1.5, and 2 mm. The final thickness of the specimens was confirmed using a digital micrometer with an accuracy of ±0.01 mm.

Before the measurements were made, all specimens were ultrasonically cleaned in distilled water for 10 min to eliminate the debris.

CIELab (Commission International de l'Eclairage *L*∗, *a*∗, *b*∗) values of each specimen were measured on a black background and on a white background with a spectrophotometer (VITA Easyshade V, VITA Zahnfabrik, Germany) in the wavelength range of visible light, 400 to 700 nm, at 10 nm intervals. D65 illuminant and 2-degree observer were selected. Prior to each measurement, the spectrophotometer was calibrated according to the manual. The probe tip was placed perpendicularly to the specimen surface. PTFE (polytetrafluorethylene) mold was used to create a dark environment. Three measurements were performed on different points of each disk by the same researcher, and the means were calculated.

TP of each specimen was obtained by calculating the color difference between the specimen against the black background and against white background using the following equation:

(1) $$\begin{array}{c} \text{TP}={\left[{\left(L_{\text{b}}*-L_{\text{w}}*\right)}^{2}+{\left(a_{\text{b}}*-a_{\text{w}}*\right)}^{2}+{\left(b_{\text{b}}*-b_{\text{w}}*\right)}^{2}\right]}^{1/2}, \end{array}$$

where *L*∗ refers to the brightness, *a*∗ to redness to greenness, and *b*∗ to yellowness to blueness. The subscripts refer to the color coordinates with, respectively, the black (b) and white (w) background. The greater the TP value, the higher the translucency of the material.

CR was measured from the luminous reflectance (*Y*) of the specimens with a black and white background. The mean CR was calculated as CR = *Y*_b_/*Y*_w_. A value of 0 shows the specimen is translucent, whereas 1 shows total opacity.

After the measurements were made, the specimens were subjected to hydrothermal degradation in an autoclave at 134°C and 0.2 MPa for 5 hours (Lisa autoclave, W&H, Austria). Then, TP and CR values were measured after the aging process.

Statistical analysis was performed for translucency values using SPSS software (version 17.0, SPSS Inc., Chicago, IL, USA). Data distribution was normal according to Kolmogorov-Smirnov. Thus, 3-way analysis of variance (ANOVA) was used to compare groups. Tukey HSD post hoc test was used to determine which group differed. The correlation between TP and CR values was analyzed using the Pearson correlation test. *p* values of <0.05 were considered significant for all analyses.

## 3. Results

Three-way ANOVA revealed that the TP values of the specimens were significantly influenced by material, thickness, and aging (*p* < 0.001). The mean TP values and standard deviations for each group are presented in Table 2. The XT and XTV groups showed significantly higher translucency values than the HT and HTV groups. Additionally, TP decreased as thickness of specimen increases regardless of the material type. The highest TP (25.84 ± .66) was obtained for the XT-0.5 mm group, while the HT-2 mm group had the lowest TP (5.04 ± .34) values.

Statistical analysis revealed that the veneered groups (XTV, HTV) showed more translucency according to the nonveneered groups (HT, XT). However, post hoc pairwise comparisons among all groups indicated that the difference in TP values between HT and HTV is statistically significant, whereas the difference between XT and XTV is not.

Differences in TP values before and after aging are presented in Table 3. Accordingly, the mean values of TP of all groups decreased after aging. The highest decrease was found in the XT-0.5 mm group (ΔTP = −4.39) while the HTV-2 mm group showed the lowest decrease after aging (ΔTP = −1.16).

Table 4 presents the mean CR values and standard deviations for each group. When analyzed using CR, the lowest value (0.38 ± 0.02) was found for XT-0.5 mm, whereas the highest CR (0.94 ± 0.03) was found in the HT-2 mm group (Table 5). In addition, CR values increased as the thickness of specimen increased in all groups.

CR difference before and after aging is presented in Table 5. It reveals that the values of CR were increased in all groups after aging. CR difference before and after aging was the highest in the XT-0.5 mm group (ΔCR = 0.09) whereas the HTV-2 mm group showed the lowest increase after aging (ΔCR = 0.03).

**Figure 2:**
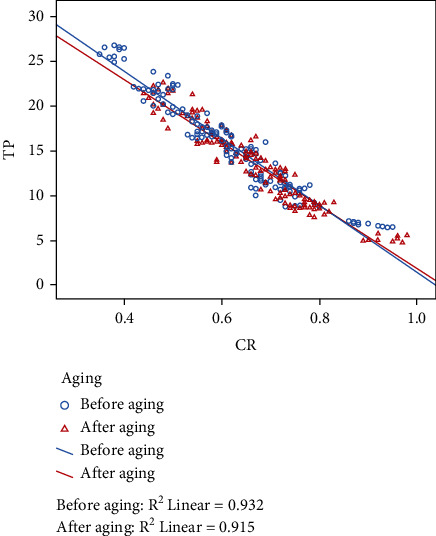
Pearson correlation analysis.

Pearson correlation test results revealed a negative correlation between TP and CR values of specimens.  Figure 2 reveals that TP increases as CR decreases after both before and after aging.

## 4. Discussion

This study investigated the effect of veneering and aging on translucency of extra and high translucent zirconia blocks with different thicknesses. Based on the results of the study, the hypothesis that type of material, veneering procedure, thickness, and aging would not affect the optical properties was rejected.

Translucency is an important factor that affects esthetics of restoration. In the present study, a spectrophotometer was used to measure the translucency. According to the CIELab color system, the translucency of the specimens was evaluated with TP and CR. TP uses color differences, and CR is a ratio of reflectance values [5].

In the current study, minimum thickness was determined to be 0.5 mm. Thickness of zirconia ceramics significantly affected its translucency, which is supported by some studies [5, 10, 23, 24]. It is clear that increasing thickness reduced light transmittance. The highest translucency was observed in the 0.5 mm thickness in VITA YZ XT.

In the present study, all specimens of VITA YZ XT showed higher translucency compared to those of VITA YZ HT. These findings are in compliance with the results of some previous studies. The results of Sen and Isler also showed significant difference between those two high-translucency zirconia blocks. This finding can be associated to larger grain size and more cubic phase of VITA YZ XT according to VITA YZ HT. Cubic phase which is optically isotropic increases the translucency by avoiding the high scattering and birefringence of light [1]. Additionally, larger grain size may cause less scattering of the incident light and higher translucency [25].

Based on the findings of our study, there is no significant difference between veneered and nonveneered specimens of VITA YZ XT blocks in the same thickness. Although it is expected much higher translucency due to the high translucency of feldspathic veneering ceramics, the reason why a significant difference is not found may be porosity between the layers and the light transmittance of the core-veneer material after firings. These factors were also mentioned in Heffernan et al.'s previous study [8]. On the contrary, there is a significant difference in VITA YZ HT blocks between nonveneered and veneered in the same thickness. It may be attributed to translucency values of VITA YZ XT and feldspathic ceramics are close to each other according to those of VITA YZ HT and feldspathic ceramics.

No glazing was performed on the specimens in this study since it could influence the final optical properties which were in agreement with previous studies. Glazing produces smooth surface and it helps increase light transmittance [26]. On the contrary, Wang et al. [27] smooth surface displayed a higher reflectance and lower transmittance and TP values.

Steam autoclave application is used for accelerated aging test method of low-temperature degradation (LTD). Hydrothermal aging was done at 134°C and 0.2 MPa for 5 h which is equivalent to 15-20 years in oral environment [28]. There was a decrease in TP values in both types of zirconia after aging. It is probably associated with t-m phase transformation of zirconia. Fathy et al. [15] reported that grain size has decreased after aging, and it was reported that larger grains are more resistant to transformation. Besides, aging increased the surface roughness which results in increasing light scattering and decreasing translucency values [29]. These results are in accordance with the other studies [15, 16, 30].

VITA YZ XT is more affected by aging compared to VITA YZ HT which might be due to larger grain size and more cubic phase of extra translucent zirconia. These findings are consistent with Fathy et al.'s study [15]. Change in TP values of translucent zirconia blocks without veneer was found higher compared to that of veneered zirconia. It can be explained by the fact that monolithic zirconia restorations are directly exposed to intraoral environment which leads to LTD. In the current study, it was found that the more decreasing the thickness, the more it is affected by the aging process. Similarly, Abdelbary et al. [21] stated that aging has a significant effect on thinner sections of zirconia. Lee et al. [31] stated that differences in TP values are 2 or greater; it is perceivable by the naked eyes. In the current study, ∆TP was found below the clinical perceptibility threshold. Also, differences in CR values are 0.07 or greater; they can be detected by the naked eye [32]. In our study, it is not clinically perceivable due to changes in CR values that are below 0.07. A correlation between TP and CR is found which is in line with Barizon et al.'s study [33].

The limitation of the study was that aging process simulates oral environment without chemical and mechanical factors. Therefore, further studies are needed to evaluate the effects of different aging conditions on optical properties of zirconia blocks to reflect clinical conditions.

## 5. Conclusions

Within the limitations of the present study, the following conclusions were reached. The translucency parameter of zirconia ceramics was significantly influenced by both material type and veneering. As thickness of specimen increases, translucency decreases. VITA YZ XT was found more translucent compared to VITA YZ HT. Veneering caused a statistically significant increase in translucency of HT, but not in XT. As specimen thickness decreases, it becomes more prone to aging. Also, extra translucent and nonveneered zirconia groups are more susceptible to hydrothermal aging.

## Figures and Tables

**Table 1:** Chemical composition and manufacturer of materials.

Material	Composition	Manufacturer
VITA YZ HT	ZrO_2_ (90-95%) Y_2_O_3_ (4-6%) HfO_2_ (1-3%) Al_2_O_3_ (0-1%) Pigments (0-1%)	VITA Zahnfabrik, Germany

VITA YZ XT	ZrO_2_ (86-91%) Y_2_O_3_ (8-10%) HfO_2_ (1-3%) Al_2_O_3_ (0-1%) Pigments (0-1%)	VITA Zahnfabrik, Germany

**Table 2:** Mean TP values and standard deviations of veneered (XTV, HTV) and nonveneered (XT, HT) specimens.

Thickness	Aging	XTV	XT	HTV	HT
0.5	Before		25.84 (0.66)^A,a^		19.43 (0.50)^B,a^
	After		21.45 (0.69)^*α*,b^		16.06 (0.45)^*β*,b^

0.5 + 0.5; 1	Before	21.98 (1.01)^C,c^	21.72 (0.40)^C,c^	17.43 (0.82)^D,c^	14.95 (0.33)^E,c^
	After	19.02 (1.16)^*γ*,d^	18.36 (0.80)^*γ*,d^	15.46 (0.93)^*δ*,d^	12.35 (0.41)^*ε*,d^

0.5 + 1.0; 1.5	Before	17.24 (0.87)^F,e^	16.82 (0.46)^F,e^	14.36 (0.66)^G,e^	10.65 (0.51)^H,e^
	After	15.04 (1.03)^*ζ*,f^	14.24 (0.49)^*ζ*,f^	12.89 (0.60)^*η*,f^	8.64 (0.36)^*θ*,f^

0.5 + 1.5; 2	Before	12.34 (1.10)^I,g^	11.63 (0.51)^I,g^	9.54 (0.93)^J,g^	6.67 (0.24)^K,g^
	After	10.51 (1.22)^*ι*,h^	9.61 (0.48)^*κ*,h^	8.38 (0.60)^*λ*,h^	5.04 (0.34)^*μ*,h^

Same superscript uppercase letters and symbols indicate no statistically significant difference within each row. Same superscript lowercase letters indicate no statistically significant difference within each column (*p* < 0.05).

**Table 3:** Difference between TP values of specimens before and after aging.

Thickness	XTV	XT	HTV	HT
0.5		-4.39 (0.67)^A,b^		-3.37 (0.49)^B,f^
0.5 + 0.5; 1	-2.96 (1.92)^C,a^	-3.36 (0.99)^C,c^	-1.97 (0.87)^C,e^	-2.60 (0.43)^C,g^
0.5 + 1; 1.5	-2.20 (1.02)^DE,a^	-2.58 (0.76)^D,cd^	-1.47 (0.96)^E,e^	-2.01 (0.43)^DE,h^
0.5 + 1.5; 2	-1.83 (1.94)^F,a^	-2.02 (0.22)^F,d^	-1.16 (0.73)^F,e^	-1.63 (0.39)^F,h^

Same superscript uppercase letters indicate no statistically significant difference within each row. Same superscript lowercase letters indicate no statistically significant difference within each column (*p* < 0.05).

**Table 4:** Mean CR values and standard deviations of veneered (XTV, HTV) and nonveneered (XT, HT) specimens.

Thickness	Aging	XTV	XT	HTV	HT
0.5	Before		0.38 (0.02)^A,a^		0.50 (0.02)^B,a^
	After		0.47 (0.02)^*α*,b^		0.58 (0.02)^*β*,b^

0.5 + 0.5; 1	Before	0.46 (0.03)^C,c^	0.48 (0.02)^C,c^	0.57 (0.03)^D,c^	0.64 (0.02)^E,c^
	After	0.52 (0.05)^*γ*,d^	0.55 (0.02)^*γ*,d^	0.62 (0.03)^*δ*,d^	0.70 (0.02)^*ε*,d^

0.5 + 1; 1.5	Before	0.57 (0.03)^F,e^	0.58 (0.02)^F,e^	0.65 (0.03)^G,e^	0.73 (0.03)^H,e^
	After	0.62 (0.03)^*ζ*,f^	0.64 (0.02)^*ζ*,f^	0.69 (0.03)^*η*,f^	0.78 (0.02)^*θ*,f^

0.5 + 1.5; 2	Before	0.68 (0.03)^I,g^	0.70 (0.02)^I,g^	0.76 (0.03)^J,g^	0.90 (0.03)^K,g^
	After	0.72 (0.02)^*ι*,h^	0.75 (0.03)^*ι*x,h^	0.79 (0.03)^*κ*x,h^	0.94 (0.03)^*λ*,h^

Same superscript uppercase letters and symbols indicate no statistically significant difference within each row. Same superscript lowercase letters indicate no statistically significant difference within each column (*p* < 0.05).

**Table 5:** Difference between CR values of specimens before and after aging.

Thickness	XTV	XT	HTV	HT
0.5		0.09 (0.02)^A,b^		0.08 (0.03)^A,f^
0.5 + 0.5; 1	0.06 (0.05)^B,a^	0.07 (0.03)^B,bc^	0.05 (0.04)^B,e^	0.06 (0.04)^B,f^
0.5 + 1; 1.5	0.05 (0.04)^C,a^	0.06 (0.02)^C,cd^	0.04 (0.03)^C,e^	0.05 (0.04)^C,f^
0.5 + 1.5; 2	0.04 (0.03)^D,a^	0.05 (0.02)^D,cd^	0.03 (0.03)^D,e^	0.04 (0.03)^D,f^

Same superscript uppercase letters indicate no statistically significant difference within each row. Same superscript lowercase letters indicate no statistically significant difference within each column (*p* < 0.05).

## Data Availability

The data used to support the findings of this study are available at Medistatistics Ltd. database.
